# Maternal uniparental disomy 14 and mosaic trisomy 14 in a Chinese boy with moderate to severe intellectual disability

**DOI:** 10.1186/s13039-016-0274-4

**Published:** 2016-08-24

**Authors:** Shujie Zhang, Haisong Qin, Jin Wang, Luping OuYang, Shiyu Luo, Chunyun Fu, Xin Fan, Jiasun Su, Rongyu Chen, Bobo Xie, Xuyun Hu, Shaoke Chen, Yiping Shen

**Affiliations:** 1 Department of Genetic and Metabolic Central Laboratory, Guangxi Maternal and Child Health Hospital, No.59, Xiangzhu Road, Nanning, China; 2 Department of Laboratory Medicine, Boston Children’s Hospital, Harvard Medical School, 300 Longwood Avenue, Boston, MA 02115 USA

**Keywords:** Maternal uniparental disomy 14, UPD(14)mat, Mosaicism, SNP array

## Abstract

**Background:**

Both maternal uniparental disomy 14 (UPD(14)mat) and mosaic trisomy 14 are rare events in live individuals. A combination of the two events in one individual is rarely encountered. Only six live-born cases have so far been reported.

**Case presentation:**

Here we reported a case of concomitant UPD(14)mat and mosaic trisomy 14 in a 10-year-old Chinese patient. Most clinical features of our patient were consistent with those previous reported for UPD(14)mat cases, which include prenatal and postnatal growth retardation, neonatal hypotonia, feeding difficulty, intellectual disability, truncal obesity, small hands and feet, short stature, and mild facial dysmorphism, but our patient showed more severe intellectual disability and no sign of precocious puberty. SNP array analysis revealed a mixture of chromosome 14 maternal isodisomy with heterodisomy and a low level trisomy mosaicism of whole chromsome 14 in blood and hyperpigmented skin samples, whereas only UPD(14)mat was detected in normal skin sample. Cytogenetic analysis identified one trisomy 14 cell in 100 metaphase of peripheral blood lymphocytes (47,XX, +14[1]/46,XX[99]).

**Conclusions:**

To our knowledge, this is the first case of a patient with UPD(14)mat and mosaic trisomy 14 reported in a Chinese patient. The definitive genetic diagnosis is beneficial for genetic counseling and clinical management of our patient, and for improving our understanding of the genotype-phenotype correlations of concomitant UPD(14)mat and mosaic trisomy 14.

**Electronic supplementary material:**

The online version of this article (doi:10.1186/s13039-016-0274-4) contains supplementary material, which is available to authorized users.

## Background

Uniparental disomy refers to the inheritance of two homologous chromosomes from one parent [1]. Maternal uniparental disomy 14 (UPD(14)mat) is a rare but clinically well-established disorder which is characterized with prenatal and postnatal growth retardation, neonatal hypotonia, feeding difficulty, motor development delay, mild to moderate intellectual disability, precocious puberty, truncal obesity, small hands and feet, short stature, hyperextensible joints, and mild facial dysmorphism [2, 3]. Since it was firstly described by Temple et al., 81 cases have been reported to date [4–11] (http://upd-tl.com/upd.html [accessed 24/07/2016]). Most UPD(14)mat cases were found with balanced Robertsonian translocations or extra structurally abnormal chromosomes (ESACs), however UPD(14)mat also occurred with normal karyotype [12, 13]. UPD(14)mat may be presented as isodisomy or heterodisomy or as a mixture of both. Both maternal iso- and heterodisomy of chromosome 14 were observed with the similar clinical findings, which indicated that the phenotypes were due to genomic imprinting rather than homozygosity for a recessive gene [12].

Trisomy 14 mosaicism is a rare chromosomal abnormality with distinct and recognizable clinical features, including growth and psychomotor retardation, short neck, congenital heart defect, genitourinary abnormalities, body asymmetry, abnormal skin pigmentation and craniofacial dysmorphism [14]. So far, 42 live-born cases have been reported [15, 16].

The first case of UPD(14)mat with mosaic trisomy 14 confirmed at peripheral blood lymphocytes level was described by Antonarakis et al. [17]. So far only six live-born patients have been previously reported [4, 17–21], four out of the six patients involved no Robertsonian translocations or ESACs. Here we reported a seventh live case of UPD(14)mat and mosaicism for trisomy 14 (the fifth case without involvement of Robertsonian translocations or ESACs).

## Case presentation

### Patient

**Fig. 1:**
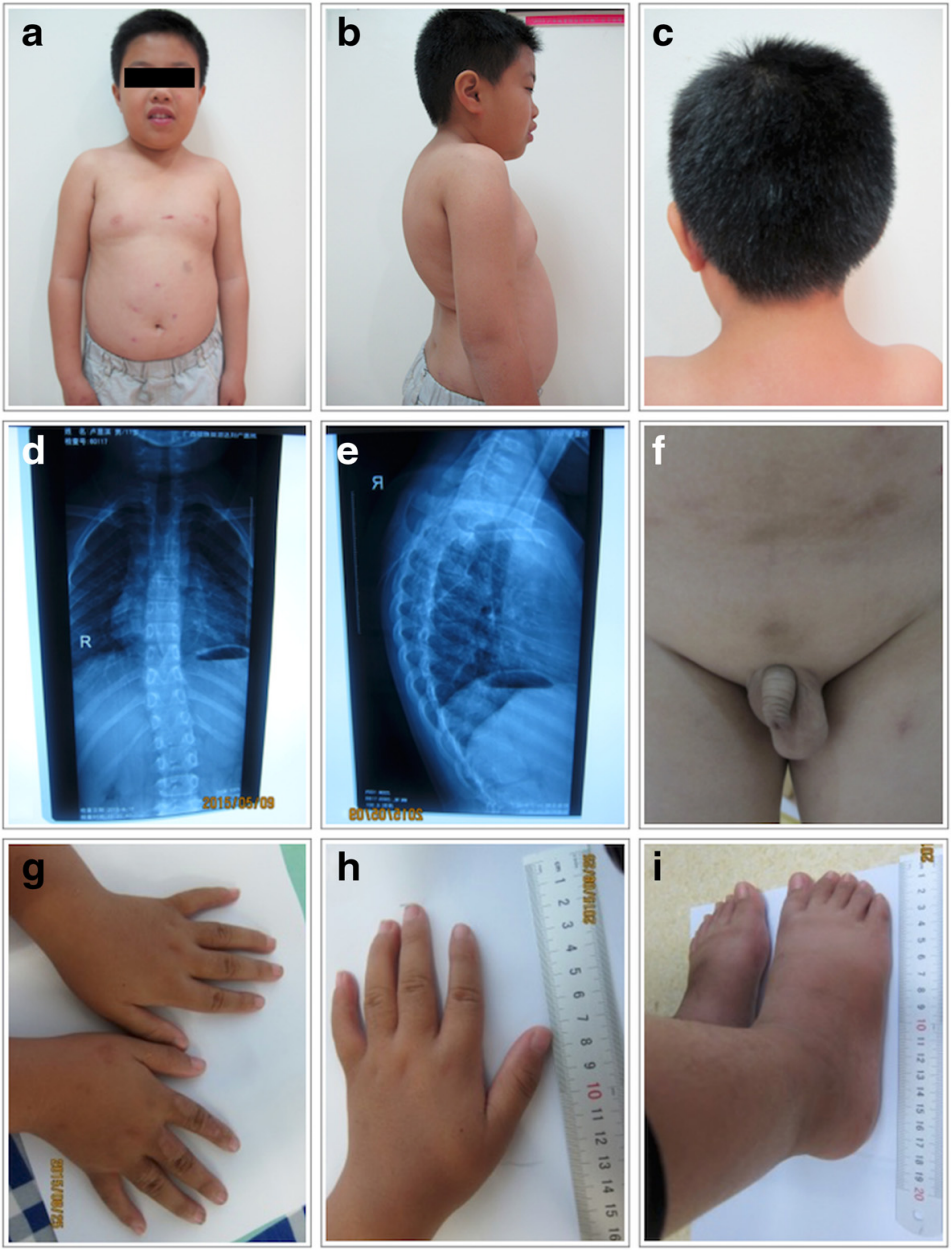
Patient at age of 9 years and 9 months. **a** Multiple facial dysmorphisms include ocular hypertelorism, narrow palpebral fissures and depressed nasal bridge. **b**, **d**, **e** Buffalo hump, thoracic-lumbar scoliosis, lordosis and kyphosis. **c** Short neck. **f** Cryptorchidism on the right side and abnormal skin pigmentation. **g**, **h** Small hands, fifth finger clinodactyly. **i** Small feet

A 10-year-old Chinese boy was born to a healthy gravida 2, para 2 mother with negative family history for genetic diseases. At the time of his birth, his mother and father were 35 and 36 years old respectively. Ultrasonic examination showed intrauterine growth retardation and polyhydramnios. He was delivered by Caesarean section at full term with a birth weight of 2350 g (3rd centile). The birth height was unknown. He had a history of developmental delay and neonatal hypotonia. He had delayed milestones, feeding difficulty and week sucking and crying during the first several months of life. At the age of five, his complete blood count and thyroid-stimulating hormone (TSH) were normal, but the serum growth hormone (GH) level was very low (0.97 ng/ml). Growth hormone provocative test showed a peak value of 2.66 ng/ml and the patient was diagnosed with growth hormone deficiency (GHD). He began to get fat with a growth rate of 2-3 cm per year when he was 6 years old and his bone age was delayed (corresponded to the age of 2 years). He is now presented with intellectual disability, language delay, learning difficulty, attention deficit and minimal communication with his peers. Physical examination at age of 9 years and 9 months showed a height of 118.3 cm (−6.0SD), a weight of 27 kg (−1.5SD) (BMI = 19.3 kg2/m), and OFC of 52 cm (50–75th percentile). He had small hands with a length of 13.5 cm and small feet with a length of 18.5 cm (The average is 20.5 cm for Chinese boys at 7–10 years of age). His physical features include short stature, truncal obesity, short neck, thoracic-lumbar scoliosis, lordosis and kyphosis, small hands and feet, fifth finger clinodactyly, buffalo hump, barrel chest, body asymmetry, cryptorchidism on the right side and abnormal skin pigmentation (Fig. 1). His craniofacial features included ocular hypertelorism, narrow palpebral fissures, high arched palate, and depressed nasal bridge. The right and left testes were 1.7*0.8*1.0 cm and 1.7*0.9*1.1 cm in size respectively. The size of penis was 4.0*1.5 cm without pubes. No sign of precocious puberty was noticed. ECG examination showed sinus cardiac arrhythmia and brain MRI examination was normal. A recent assessment using Chinese Wechsler intelligence scale for children (C-WISC) showed a verbal scale IQ of 35 (severe), a performance scale IQ of 55 (mild), and a full scale IQ of 38 (severe). Raven's Standard Progressive Matrices (RSPM) was also used to measure his intelligence. The intelligence percentile rank was 6 %. Based on the intelligence test results above, the patient was diagnosed with moderate to severe intellectual disability. Meanwhile his motor development was close to normal level. The boy started growth hormone (GH) therapy at the age of 9 years and 9 months. He grew 4.9 cm (from 118.3 to 123.2 cm (−5.0SD)) during the first five months. He also gained 5 kg of weight (from 27 to 32 kg (−0.5SD)).

### Cytogenetic and molecular analyses

Peripheral blood lymphocytes, hyperpigmented and normal skin samples were collected from the patient and genomic DNA was extracted using Lab-Aid DNA kit (Zeesan Biotech Co., Ltd, China). G-banding was performed on metaphase chromosomes of cultured peripheral blood lymphocytes of the family trio. FISH was directly conducted on interphases chromosomes 14 of peripheral blood lymphocytes with probe specific for the region 14q11.2 (Agilent, USA, Spectrum red) on the patient. Genomic profiling were performed on blood, hyperpigmented and normal skin samples of the patient and his parents’ blood samples using Illumina Human CytoSNP 12 BeadChip array (Illumina, San Diego, CA). The SNP array data was analyzed with GenomeStudio and KaryoStudio software. All operative procedures fully followed the manufacturer’s instructions.

## Results

**Fig. 2:**
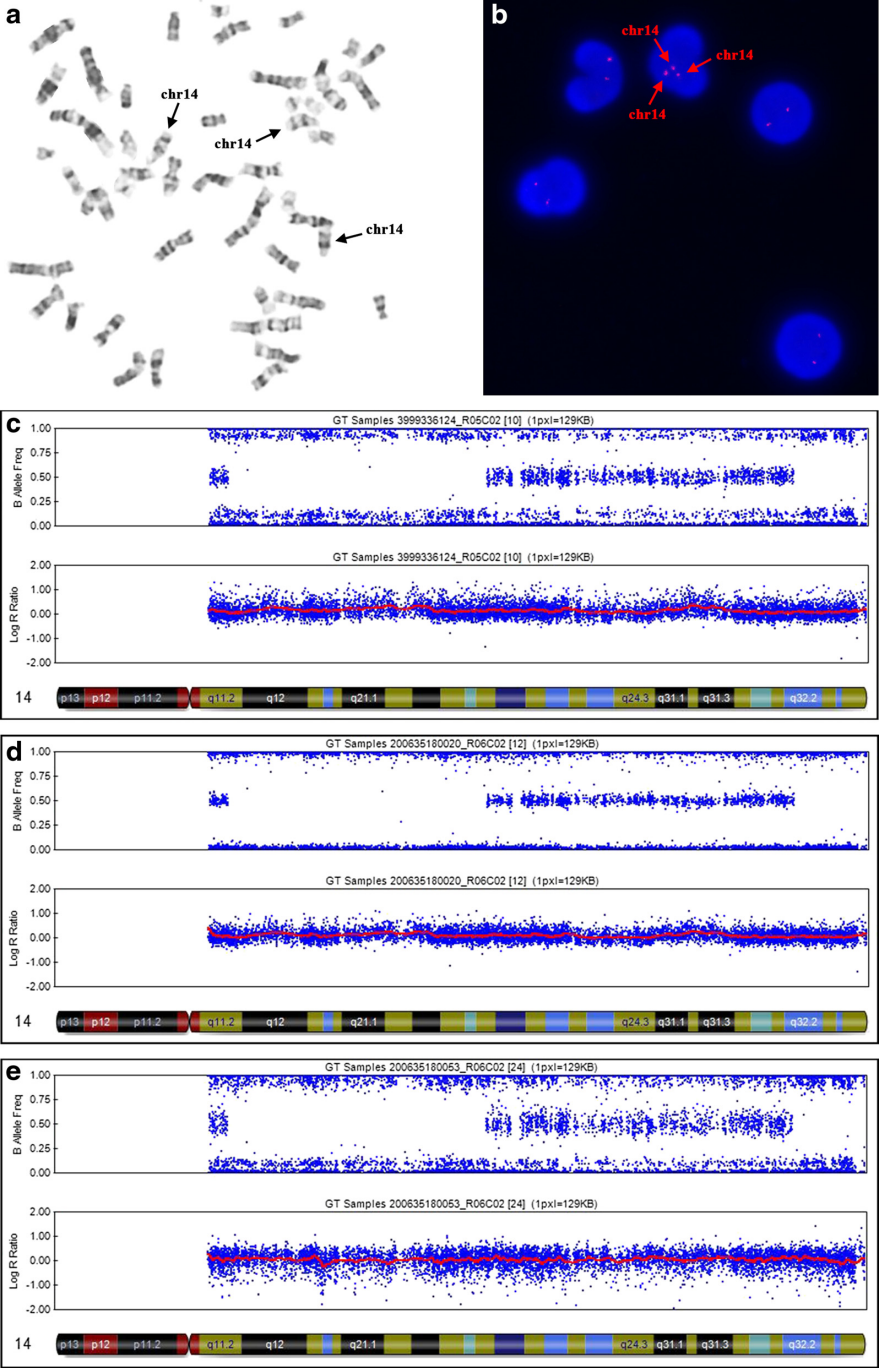
Cytogenetic and molecular results. **a** G-banding karyotype of the patient, showing a trisomy 14 cell detected from 100 metaphase cells. **b** FISH results of analysis of interphase cells. **c**, **e** Chromosomal microarray analysis on blood and hyperpigmented skin samples showed two AOH regions at 14q11.2q22.3 and 14q32.2qter and trisomy 14 mosaicism at about 10-20 % level, respectively. **d** Chromosomal microarray analysis on normal skin sample only showed two AOH regions at 14q11.2q22.3 and 14q32.2qter

G-banding karyotyping found one trisomy 14 cell in 100 metaphase cells (47,XX, +14[1]/46,XX[99]) (Fig. 2a) in the patient’s peripheral blood sample and the parents’ karyotypes were normal. The SNP array analysis revealed a low level (10–20 %) trisomy 14 mosaicism in blood and hyperpigmented skin samples (Fig. 2c, e) but not in the normal skin sample (Fig. 2d). It also revealed two segments of absence of heterozygosity (AOH) on chromosome 14, one at 14q11.2q22.3 (chr14:22749742–57116759, NCBI build 37) and another at 14q32.2qter (chr14:97636474–107282437, NCBI build 37), suggestive of segmental uniparental disomy in all types of samples (Fig. 2c–e). To confirm, we performed a parent–child trio genotyping by SNP array using blood samples of the family and all informative SNP data revealed that 14q11.2q22.3 and 14q32.2qter segments were maternal isodisomic, and the other regions of chromosome 14 were maternal heterodisomic (Additional file 1). The patient had a mixture of isodisomy and heterodisomy on chromosome 14. Thus we concluded that our patient had tissue restricted UPD(14)mat and mosaicism for trisomy 14. Subsequent peripheral blood lymphocyte inter-phases FISH showed 22 trisomy 14 cells from 200 cells (47,XX,+14[22]/46,XX[178]) (Fig. 2b).

## Discussion

This is the seventh liveborn reported with a combination of UPD(14)mat and mosaic trisomy 14 and the first case in a Chinese patient. The presence of mosaic trisomy suggest that UPD was formed by trisomy rescue. The finding of tissue restricted trisomy mosaic pattern further suggested somewhat delayed post-zygotic trisomy rescue. In addition, the UPD(14)mat presented as a mixture of isodisomy and heterodisomy in our patient, we speculated that at least two crossover events had happened between the two non-sister chromatids of homologous chromosomes at the maternal meiosis I and a disomic gamete arose from nondisjunction in maternal meiosis II [12].

Most of the clinical features of our patient were consistent with those previous reported for UPD(14)mat cases, which included intrauterine growth retardation, neonatal hypotonia, short stature, truncal obesity, small hands and feet, intellectual disability, and mild facial dysmorphism, but our patient showed more severe intellectual disability. In previous reports, most cases had normal intelligence and no more than one-third of cases showed mild to moderate intellectual disability, whereas our patients presented with moderate to severe intellectual disability [3]. In addition, our patient also presented with some clinical features of trisomy 14 mosaicism which include polyhydramnios, abnormal skin pigmentation, short neck and cryptorchidism. We compared the clinical features of five patients with UPD(14)mat and mosaic trisomy of whole chromsome 14 in Table 1 (Column 4–8). We limited the comparison among live cases and excluded two cases with the involvement of Robertsonian translocation [17, 18]. All cases presented hypotonia. IUGR, short stature and truncal obesity were consistent features. It is unclear if these features were present in the patient reported by Pecile et al. [21]. Moderate intellectual disability was described in two patients reported by Balbeur et al. and Cox et al. respectively [4, 20]. The mental status was not mentioned for the other two previously reported patients [19, 21]. Based on the C-WISC and RSPM evaluation, our patient was determined to have moderate to severe intellectual disability. No consistent or recognizable facial feature was identified among the five patients. In addition, we compared the clinical features of the five patients with the summaried features of patients with only UPD(14)mat (Column 2) or only trisomy 14 mosaicism (Column 3). It is noticed that the shared features among the five patients are also consistent features in patients with UPD(14)mat. There are few shared features between the five patients and the patients with trisomy 14 mosaicism. The result of comparision indicated that the clinical consequence of patients with concomitant UPD(14)mat and trisomy 14 mosaicism is likely largely dictated by the clinical consequence of maternal UPD.

**Table 1:** Summary of the clinical features in UPD(14)mat or Trisomy 14 mosaicism and the clinical features of liveborn previously reported with UPD(14)mat and trisomy 14 mosaicism

Clinical Category	UPD(14)mat [23]	Trisomy 14 mosaicism [16, 24]	[4]	[21]	[19] patient A	[20]	Present case
Gender/Race	NA	NA	F/NA	F/NA	F/Dutch	F/Guinean	M/Chinese
Age at Diagnosis	NA	NA	5y	2y	8.9y	15y	10y
Mother age	NA	NA	NM	NM	NM	NM	35y
Mosaic level	NA	NA	Low level	NM	Paternal chr14 in some cells	T14 = 2-49 %	T14 = 15-20 % (SNP array) T14 = 1 % (100 cells)
De Novo	NA	NA	NM	NM	NM		Y
Prenatal Manifestation	Premature birth (12/30)	Polyhydramnios (7/24)	NM	NM	38 weeks	Full term	Full term; Polyhydramnios;
Growth	Intrauterine growth retardation (IUGR) (23/29); Low birth weight (24/28); Short stature (30/37); Truncal obesity (15/30);	Growth retardation (17/24)	IUGR; 1,870 g (<3rd); Short stature; Truncal obesity;	NM	IUGR; 2,340 g(<3rd); Short stature; Truncal obesity;	IUGR; 2,000 g(<3rd); Short stature; Truncal obesity;	IUGR; 2,350 g (<3rd); Short stature; Truncal obesity;
Head and Neck	Relative macrocephaly (38/68)	Microcephaly (10/40); Short neck(14/24);	NM	NM	NM		Short neck
Face	Frontal bossing (11/40); Short philtrum (7/40); Micrognathia (7/40); Recurrent otitis media (8/40); Ocular hypertelorism; Broad nose (5/40); Depressed nasal bridge (in some patients); Anteverted nares (in some patients); High arch palate (11/40);	Frontal bossing (12/24); Abnormal palpebral fissures(15/24); hypertelorism(8/24); Palpebral ptosis(4/24); Nose abnormalities(16/24); Anteverted nostrils(4/24); Ears abnormalities(14/24); Mouth abnormalities (32/40); High arch palate(8/24); Micro/retrognathia(18/24);	Upslanting palpebral fissures; Anteverted nares; Narrow palate;	Micrognathia; Low-set ears; Hypertelorism;	Supraorbital fullness; Square and low-set ears; Small up-turned nose; Open-mouth appearance; Recurrent ear infections;	Broad and depressed nasal bridge; Short nose; Upslanting palpebral fissures;	Ocular hypertelorism; Small palpebral fissures; Depressed nasal bridge;
Gastrointestinal	Feeding difficulty (16/40)	-	Feeding difficulty	NM	NM	Feeding difficulty	Feeding difficulty
Cardiovascular	-	Congenital heart defect (24/40)	NM	Atrial septal defect	NM	NM	-
Genitourinary	Cryptorchidism (rare); Small testes (rare);	Micropenis(6/9), Cryptorchidism(7/9)	NM	NM	NM	NM	Cryptorchidism on the right side
Skeletal	Hyperextensible joints (9/15); Joint contractures (4/51); Scoliosis (6/23); Small hands (25/30) and feet (19/20); Clinodactyly (6/40);	Body asymmetry	Small hands and feet	Left hand agenesis	Loose hand joints;	Hyperextensible joints	Thoraco-lumbar scoliosis; Buffalo bump; barrel chest; Small hands and feet; Inapparent fifth finger;
Neurologic	Hydrocephalus; Hypotonia (29/32); Motor development delay (26/32); Speech delay (9/20); Mild to moderate intellectual disability (11/26); Fine motor/coordination problems (3/40);	Hypotonia (8/40); Psychomotor retardation; Seisure(5/24); Dandy–Walker malformation;	Hypotonia; language delay; Learning difficulty; Moderate degree developmental disability;	Hypotonia	Hypotonia; Motor development delay; Speech development delay;	Moderate intellectual impairment; Severe learning difficulties; Hypotonia;	Hypotonia; Learning difficulty; Attention deficit; Less communication with his peers; language delay; Moderate to severe intellectual disability; WISC:35-55-38; RSPM:6 %;
Endocrine Features	Early onset/Premature puberty (13/15); Maturity-onset diabetes of the young (rare);	-	Early puberty	NM	-	Precocious puberty;	-
Laboratory Abnormalities	Hypercholesterolemia (4/40); Hypertriglyceridemia (rare);	-	NM	NM	NM	Hypercholesterolemia	Low serum GH level
Skin	-	Abnormal skin pigmentation(11/24)	NM	NM	NM	Abnormal skin pigmentation	Abnormal skin pigmentation

*F* female, *M* male, *NA* not applicable, *NM* not mentioned; −: feature absent

The cases of concomitant UPD(14)mat and partial trisomy 14 mosaicism were not included

Interestingly, both UPD(14)mat and trisomy 14 mosaicism were detected in the hyperpigmented skin of our patient whereas only the UPD(14)mat was detected in his normal skin. Balbeur et al. detected a similar low level trisomy 14 mosaicism in both hyperpigmented and normal skin [20]. Thus it is not known if trisomy 14 mosaicism directly contributed to the abnormal skin pigmentation phenotype.

UPD(14)mat patients demonstrated overlapping features with Prader-Willi syndrome (PWS) including hypotonia, neonatal feeding difficulty and obesity. UPD(14)mat should be considered as a differential for patients with suspected PWS. Indeed, Mitter et al. detected four UPD(14)mat from 33 patients who were suspected to have PWS [9]. Similarly, Hosoki et al. identified four UPD(14)mat patients from a 78 patient cohort with PWS-like phenotype without known molecular defects for PWS [10]. However, Cox et al. did not find any UPD(14)mat in 35 patients suspected with PWS [4]. Further studies could help to identify other distinguishing features such as facial characteristics and precocious puberty for differential diagnosis.

Four UPD(14)mat patients had been previously described with GH treatment, and all patients showed beneficial effects. But only one patient was known to have growth hormone deficiency [9], and other three cases were treated because of short stature without data regarding the GH level [19, 22]. The height SDS (HSDS) of patient with growth hormone deficiency increased from −2.5SD at age of 6 to −1.5SD at age of 12 [9]. Two patients presented a considerable increase in height (from −2.3SD at age of 6.9 to −1.2SD at age of 8.9, from −1.2SD at age of 9.3 to −0.6SD at age of 11.4, respectively) and IGF-1 level (from +0.1SD to +1.3SD, from −1.4SD to +0.9SD, respectively) [19]. The remaining patient received growth hormone therapy at age of 4 because of short stature (−3.9SD at 3 years 11 months) and obtained effective result without specific data about height [22]. The treatment effect on body composition was less consistent among them. In one patient, her weight decreased from +1.2SD to −0.7SD and the body composition was improved (fat percentage from 51.5 % to 45.4 %), and in other patient, his weight and body composition remained stable [19]. The GH level was rarely measured among the UPD(14)mat patients, our patient was the second case to undergo growth hormone provocative test. Due to the complete growth hormone deficiency, our patient started the recombinant human growth hormone replacement treatment at the age of 9 years and 9 months. After five months of treatment, his height increased from −6.0SD to −5.0SD. Contrary to previous reports, his weight increased from −1.5SD to −0.5SD which may be caused by other unknown endocrine problems or overeating behavior. The clinical presentation described for this patient and his response to GH treatment is useful for future patient counseling and care.

## Conclusions

In summary, we described the first Chinese patient with UPD(14)mat and mosaic trisomy 14. Our patient presented with prenatal and postnatal growth retardation, neonatal hypotonia, feeding difficulty, intellectual disability, short stature, truncal obesity, small hands and feet, and mild facial dysmorphism, mostly consistent with features known to be associated with UPD(14)mat. The patient had complete GH deficiency and benefited for growth hormone replacement treatment. The definitive genetic diagnosis is beneficial for genetic counseling and clinical management of our patient, and for improving our understanding of the genotype-phenotype correlations of concomitant UPD(14)mat and mosaic trisomy 14.

### Consent

Written informed consent was obtained from the parents of the patient for publication of this Case report and any accompanying images. The study was approved by the ethical committee of Guangxi Maternal and Child Health Hospital, China. A copy of the written consent is available for review upon request.

## Abbreviations

AOH, absence of heterozygosity; C-WISC, Chinese Wechsler intelligence scale for children; ESACs, extra structurally abnormal chromosomes; FISH, fluorescent in situ hybridization; GH, growth hormone; GHD, growth hormone deficiency; PWS, Prader-Willi syndrome; RSPM, Raven’s standard progressive matrices; SNP array, single nucleotide polymorphism array; TSH, thyroid-stimulating hormone; UPD(14)mat, maternal uniparental disomy 14.

## Additional file

Additional file 1: SNP array raw data for chromosome 14 of the family trio. (XLS 1028 kb)
